# The Role of Neutrophil-to-Lymphocyte Ratio (NLR) in Urosepsis-Associated Delirium

**DOI:** 10.7759/cureus.62110

**Published:** 2024-06-10

**Authors:** Alice Nicoleta Dragoescu, Vlad Padureanu, Andreea Doriana Stanculescu, Luminita Chiutu, Rodica Padureanu, Maria Andrei, Mihai Alexandru Radu, George Mitroi, Petru Octavian Dragoescu

**Affiliations:** 1 Department of Anesthesiology and Intensive Care, University of Medicine and Pharmacy of Craiova, Craiova, ROU; 2 Department of Internal Medicine, University of Medicine and Pharmacy of Craiova, Craiova, ROU; 3 Department of Cardiology, Emergency Clinical County Hospital of Craiova, Craiova, ROU; 4 Department of Urology, Emergency Clinical County Hospital of Craiova, Craiova, ROU; 5 Department of Urology, University of Medicine and Pharmacy of Craiova, Craiova, ROU

**Keywords:** prevention, management, diagnostic, intensive care unit, septic shock [ss], sepsis, urosepsis-associated delirium, neutrophil-to-lymphocyte ratio (nlr)

## Abstract

Introduction

Urosepsis is a systemic, dysregulated, inflammatory reaction to a urinary tract infection and can have severe effects on all systems, which can often lead to multi-organ failure and death. Sepsis-associated delirium is a common complication in critically ill patients, contributing to adverse outcomes and prolonged hospital stays. The neutrophil-to-lymphocyte ratio (NLR) has emerged as a potential biomarker for sepsis severity and prognosis.

Material and methods

Our study investigates the utility of NLR in the diagnostic strategies for urosepsis-associated delirium in a cohort of 76 patients with sepsis and septic shock admitted to the Intensive Care Unit (ICU). We performed a single-centre retrospective observational study in the Craiova Clinical Emergency Hospital between June and October 2023.

Results

Patients with urological conditions that were diagnosed with urosepsis included 76 patients. These patients were clustered as follows: a group with delirium (37 patients, 48.7%) and another group without delirium (39 patients, 51.3%). Complete blood count parameters were obtained upon admission, and delirium was assessed using standardized diagnostic criteria. We identified a strong significant positive correlation between elevated NLR values on ICU admission and the development of delirium during hospitalization in urosepsis patients. Receiver operating characteristic (ROC) analysis showed similar diagnostic performance for NLR score.

Conclusions

The findings suggest that NLR may serve as a valuable biomarker for early detection, risk stratification, and guiding therapeutic interventions in urosepsis-associated delirium, thus improving outcomes in critically ill patients.

## Introduction

Sepsis is one of the most common causes of morbidity and mortality around the world. Sepsis is a potentially life-threatening condition caused by a systemic dysregulated host response to infection. The central nervous system is particularly susceptible to dysfunction caused by sepsis, neurological signs including confusion, agitation, lethargy, or coma, and symptoms of sepsis-associated delirium [1]. The term “delirium” is used globally instead of “encephalopathy” in current diagnostic manuals such as the Diagnostic and Statistical Manual of Mental Disorders (DSM-5) and the International Classification of Diseases (ICD-10).

Delirium is characterised by an acutely altered mental status accompanied by reductions in cognitive function and attention. Delirium occurs in 20-40% of critically ill patients [2-4] and sepsis is one of the most important risk factors for delirium [1,5,6]. Urosepsis-associated delirium is a significant complication in critically ill patients admitted to the intensive care unit (ICU), characterised by acute alterations in mental status. Sepsis-associated delirium (SAD) is described by a modified level of consciousness with a decreased ability to maintain, or shift attention associated with either a modification in cognition or the increase of a perceptual disturbance [1]. SAD is considered a diffuse cerebral dysfunction caused by the systemic inflammatory response to an infection in the absence of any signs of infection in the central nervous system [7].

Sepsis and delirium are associated with increased morbidity and mortality [8-10], so it is important to promptly early diagnose and treat sepsis-associated delirium [8,10,11]. The actual clinical practice guidelines on delirium from the Society of Critical Care Medicine (SCCM) recommend regularly assessing for delirium using a validated tool such as the Confusion Assessment Method-ICU (CAM-ICU) or the Intensive Care Delirium Screening Checklist (ICDSC) [12-15].

Multiple studies showed that CAM-ICU has a higher sensitivity (80%) and specificity (95.9%) than the ICDSC (sensitivity 74% and specificity 81.9%) [16]. The CAM-ICU scale helps detect delirium by evaluating four key features [16,17]: 1. Acute onset or fluctuating course: Determining whether there's a sudden change in mental status or if it fluctuates throughout the day, 2. Inattention: Assessing the patient's ability to maintain attention by performing tasks like identifying letters or digits in a sequence, 3. Disorganized thinking: Evaluating the coherence and logical flow of the patient's thoughts by asking questions or observing their responses, 4. Altered level of consciousness: Checking for any signs of altered consciousness such as drowsiness or reduced awareness of surroundings.

Diagnostic of sepsis-associated delirium features include an acute change or fluctuation in mental status (feature 1) and inattention (feature 2), and one of the following: (a) disorganized thinking (feature 3) or (b) altered level of consciousness (feature 4) [16]. The diagnosis of delirium using the CAM-ICU involves a non-sedated patient with a Richmond Agitation-Sedation scale (RASS) score of -3 or lighter [11].

The neutrophil-to-lymphocyte ratio (NLR) is a valuable assessment in sepsis, reflecting the balance between the inflammatory response and immune suppression. Elevated NLR has been associated with increased severity and mortality in septic patients [18,19] and is considered a promising biomarker for sepsis severity and prognosis [20]. Our objective for this study is to evaluate the role of NLR in the diagnosis and severity assessment of urosepsis-associated delirium.

## Materials and methods

This is a single-centre prospective observational study performed in the Craiova Clinical Emergency Hospital. Ethics Committee approval no. 79/07.04.2023 was obtained prior to study initiation. We included 76 consecutive patients admitted for urinary sepsis in our hospital in a six-month timeframe, between June and November 2023 within an academic research grant. We diagnosed urinary sepsis and septic shock according to Sepsis-3 criteria as follows: sepsis - suspected or documented urinary tract infection and SOFA score ≥ 2; septic shock - sepsis plus vasopressor therapy needed to maintain MAP ≥ 65 mmHg plus lactate ≥ 2 mmol/L (18 mg/L) despite adequate fluid resuscitation [8].

Inclusion criteria for the study were: age ≥ 18 years, sepsis diagnosed by the National Early Warning Score (NEWS) and/or Sequential Organ Failure Assessment (SOFA) scores as well as a urinary tract condition (pyelonephritis, obstructive uropathy, stone disease, pyonephrosis, indwelling urinary catheters, etc.) or a recent endoscopic or percutaneous urological procedure. Exclusion criteria were: subjects younger than 18 years old, pregnant females, patients with compromised immunity, advanced cancers or other terminal illnesses, a history of mental illness or dementia, using antipsychotic medications, alcohol abuse, drug abuse, inapt for good communication. Before study enrolment, patients or close relatives were informed about the study and provided signed informed consent. All patients were admitted to the ICU as soon as possible after hospital admission or after the minimally invasive urologic procedure and discharged upon symptom improvement or death.

Most patients underwent emergency urological procedures intended to decompress and drain the urinary tract, such as ureteral JJ stent placement or replacement, suprapubic or urethro-vesical catheterisation, ultrasound-guided percutaneous nephrostomy or perirenal drainage. The majority of the patients received local or regional/spinal anaesthesia, and only seven patients received general anaesthesia with laryngeal mask. Medical history, as well as clinical signs and symptoms and vital signs, were collected from all patients. Peripheral venous blood sampling was drawn immediately after admission for standard blood tests, biochemistry, and serum lactate, as well as blood inflammation markers: C-reactive protein (CRP) and procalcitonin (PCT). Before starting the empiric antibiotic, two pairs of blood cultures (aerobic and anaerobic) as well as urinalysis and urine culture were collected. We calculated the NLR value at admission, which is known to normally range between 1 and 3 [19]. C-reactive protein (CRP) values were measured using immuno-turbidimetry method. Procalcitonin was analysed on Elecsys Cobas e601 Roche® (Roche Diagnostics, USA), which is a fully automated analyser that uses the electro-chemiluminescence immunoassay (ECLIA) principle. It is designed for both quantitative and qualitative in vitro assay determinations. The NEWS and SOFA scores were calculated upon admission to the hospital. All patients were assessed by abdominal ultrasound and abdominal CT scan for urinary tract evaluation.

For diagnostic criteria for delirium, we used the Confusion Assessment Method in Intensive Care Unit (CAM-ICU) and RASS criteria [17] (Tables 1, 2). All patients were assessed for delirium once daily during ICU stay. Pain intensity was also assessed on a daily basis using the Visual Analog Scale (VAS).

**Table 1:** The Confusion Assessment Method for the Intensive Care Unit (CAM-ICU) RASS - Richmond Agitation Sedation Scale, GCS - Glasgow Coma Scale Reference No. [17]

Items	Grading	Score
1. Acute Onset or Fluctuating of Mental Status Is the pt different than his/her baseline mental status? Or Has the patient had any fluctuation in mental status in the past 24 hours as evidenced by fluctuation on a sedation scale (i.e., RASS), GCS, or previous delirium assessment?	0 – absent 1 - present	-
2. Inattention Say to the patient, “I am going to read you a series of 10 letters. Whenever you hear the letter ‘A,’ indicate by squeezing my hand.” Read letters from the following letter list in a normal tone 3 seconds apart. S A V E A H A A R T (Errors are counted when patient fails to squeeze on the letter “A” and when the patient squeezes on any letter other than “A.”)	0 – absent (correct >8) 1 – inattention (correct 4-7) 2 – severe inattention (correct 0-3)	-
3. Altered Level of Consciousness Present if the Actual RASS score is anything other than alert and calm (zero)	0 – absent (RASS 0) 1 – altered level (RASS 1, -1) 2 – severe altered level (RASS >1, < -1)	-
4. Disorganized Thinking Yes/No Questions: 1. Will a stone float on water? 2. Are there fish in the sea? 3. Does one pound weigh more than two pounds? 4. Can you use a hammer to pound a nail? Errors are counted when the patient incorrectly answers a question. Command: Say to patient: “Hold up this many fingers” (Hold 2 fingers in front of patient) “Now do the same thing with the other hand” (Do not repeat number of fingers) * If patient is unable to move both arms, for 2 nd part of command ask patient to “Add one more finger” An error is counted if patient is unable to complete the entire command.	0 – absent (correct > 4) 1 – disorganized thinking (correct 2, 3) 2 – severe disorganized thinking (correct 0, 1)	-
Overall CAM-ICU 1 plus 2 and either 3 or 4 present = CAM-ICU positive	Criteria Met	CAM-ICU Positive (Delirium Present)
	Criteria not Met	CAM-ICU Negative (No Delirium)

**Table 2:** Richmond agitation-sedation scale (RASS) Reference No. [17]

Score	Term	Description	
+ 4	Combative	Overtly combative or violent; immediate danger to staff	
+3	Very agitation	Pulls on or removes tube(s) or catheter(s) or has aggressive behaviour toward staff	
+2	Agitated	Frequent non-purposeful movement or patient–ventilator asynchrony	
+1	Restless	Anxious or apprehensive but movements not aggressive or vigorous	
0	Alert and calm	Alert and calm	
-1	Drowsy	Not fully alert, but has sustained (more than 10 seconds) awakening, with eye contact, to voice	
-2	Light sedation	Briefly (less than 10 seconds) awakens with eye contact to voice	
-3	Moderate sedation	Any movement (but no eye contact) to voice	
-4	Deep sedation	No response to voice, but any movement to physical stimulation	
-5	Unarousable	No response to voice or physical stimulation	

Demographic data, clinical characteristics, laboratory parameters including NLR, and outcomes were collected from medical records. The association between NLR and the development of delirium, as well as its impact on clinical outcomes, was assessed using statistical analyses. The normality of data samples was assessed by the Kolmogorov-Smirnov test. Normally distributed data was analysed using the Student t-test, while the non-parametric analysis was performed using the Mann-Whitney U test. The Pearson r correlation coefficient was used for clinical correlations evaluation while the ROC curve analysis was employed for diagnostic performance assessment. The significance level was established at .05 for all statistical tests. Statistical analyses were performed using MedCalc software for Windows, version 22.013 (MedCalc Software, Ostend, Belgium).

## Results

Depending on their mental status, these 76 patients with urosepsis were divided into a group with delirium (37 patients, 48.7%) and another group without delirium (39 patients, 51.3%). Clinical and biological parameters, as well as, sepsis scores and markers, are presented in Table 3. The average patient age was 63.4 years and significantly higher for patients with delirium (72.7 vs 54.5 years, p < 0.001) as expected. Most of the patients were males (68.4%, 52/76 subjects), but no significant difference was found between the two sexes regarding the presence of delirium. There were significantly more patients with delirium among the patients with septic shock than those without (29.7% vs 10.2%, p <0.05).

The average ICU length of stay (LOS) was eight days and significantly longer for patients with sepsis-associated delirium (9.0 vs 7.2, p < 0.001), while overall LOS was 12 days. Delirium was also not significantly related to any of the urological conditions or complicating factors found to be associated with urosepsis (ureteral obstruction, hydronephrosis, urinary tract stones, indwelling ureteral stents or percutaneous nephrostomy catheters and history of recent endourologic surgery) or with the most frequently associated non-urological conditions (diabetes mellitus and high blood pressure).

All urological procedures intended to decompress and drain the urinary tract were minimally invasive (ureteral JJ stent placement or replacement, suprapubic or urethro-vesical catheterisation, ultrasound-guided percutaneous nephrostomy or perirenal drainage) and brief, with an average duration 19 ± 6 minutes. Also, these kinds of procedures are not usually considered as surgery. Therefore, the influence of these minimally invasive procedures on cognitive status is negligible.

Moreover, most of the procedures were performed under local or spinal anaesthesia (lignocaine 1-2% or bupivacaine 0.5%) which is known to have minimal effect on cognitive status. Only seven patients received general anaesthesia with Laryngeal Mask (propofol 1.5 mg/kg and balanced analgesia with fentanyl 1 µg/kg, nefopam 20 mg, and paracetamol 1 g), but none of these patients developed delirium during their ICU stay. 

Following daily pain intensity assessment by visual analogue scale (VAS), all patients had an average VAS score below 5 (mild-moderate pain) with no significant difference between the two groups. Thus, for all patients, pain management included multimodal analgesia with non-opioid analgesics, such as paracetamol, nefopam, nonsteroid anti-inflammatory drugs (NSAIDs), and corticosteroids.

There were no significant differences between the two groups regarding vital signs (mean arterial pressure, heart rate, respiratory rate), Glasgow coma score, haematology tests (white blood cells, neutrophils, lymphocytes, platelets, etc.), biochemistry tests (lactate, creatinine, bilirubin, etc.), microbiology results (positive urine and blood cultures) or even inflammation markers (erythrocyte sedimentation rate (ESR), CRP, procalcitonin). No significant relation with delirium was identified for any of the two sepsis scores (NEWS and SOFA).

**Table 3:** Patient demographic and biological parameters and scores with a comparison between those with sepsis and septic shock SOFA - Sequential Organ Failure Assessment, NEWS - National Early Warning Score, GCS – Glasgow Coma Scale, MAP – mean arterial pressure, HR – heart rate, RR – respiratory rate, WBC – white blood cells, NEU – neutrophils, LYM – lymphocytes, NLR – neutrophil-to-lymphocyte ratio, PLT – platelet, ESR – erythrocyte sedimentation rate, LOS – Length of Stay. Data presented as mean and standard deviation, ratio or median and inter-quartile range depending on data type and distribution (statistical tests: *= Student t-test, # = Chi-square test, ^= Mann-Whitney U test).

Parameter	Total (n=76)	Delirium (n= 37)	No Delirium (n=39)	p=
Age (years)	63.4 ±15.1	72.7 ±7.2	54.5 ±15.4	< 0.001*
Sex (M/F)	52/24	27/10	25/14	0.434#
SOFA score	6.1 ± 3.2	6.0 ± 3.1	6.2 ± 2.9	0.353*
NEWS	9.8 ± 2.9	9.6 ± 3.2	10.0 ± 2.7	0.274*
GCS	12.4 ±1.7	12.1 ±1.5	12.7 ±1.9	0.053*
MAP (mmHg)	80 ± 11	79 ± 12	80 ± 9	0.278*
Septic Shock	15/61	11/37	4/39	0.033#
HR (beats/min)	82 ± 18	84 ± 21	81 ± 16	0.272*
RR (breaths/min)	23 ± 7	23 ± 7	22 ± 7	0.331*
WBC (x103/mm3)	18 (15-22)	17 (14-20)	21 (19-23)	0.412^
NEU (x103/mm3)	15 (13-18)	14 (12-17)	18 (15-19)	0.313^
LYM (x103/mm3)	1.6 (1.2-2.1)	1.6 (1.3-2.1)	1.5 (1.2-1.8)	0.596^
NLR	10.3 ± 3.2	11.9 ± 3.4	8.7 ± 2.0	< 0.001*
PLT (x103/mm3)	133 ± 49	130 ± 47	138 ± 50	0.237*
C-Reactive Protein (mg/l)	124 (92-145)	124 (94-143)	136 (79-153)	0.697^
Procalcitonin (PCT) (ng/ml)	13.0 ±5.7	13.8 ±6.7	12.3 ±4.7	0.143*
ESR (mm/h)	40.4 ±16.4	41.8 ±16.7	39.0 ±16.2	0.228*
Lactate (mmol/l)	1.8 ± 1.4	1.8 ± 1.5	1.8 ± 1.3	0.456*
Creatinine (mg/dl)	1.9 ± 1.3	1.8 ± 1.3	2.1 ± 1.5	0.249*
Bilirubin (mg/dl)	1.8 ± 1.0	1.7 ± 0.9	1.9 ± 1.1	0.172*
ICU LOS	8.1 ± 1.8	9.0 ± 1.4	7.2 ± 1.7	< 0.001*
Overall LOS	12.0 ± 3.4	13.2 ± 3.6	10.9 ± 2.8	< 0.01*
Deaths (%)	5 (6.6%)	4 (10.8%)	1 (2.6%)	0.147^#^

**Figure 1:**
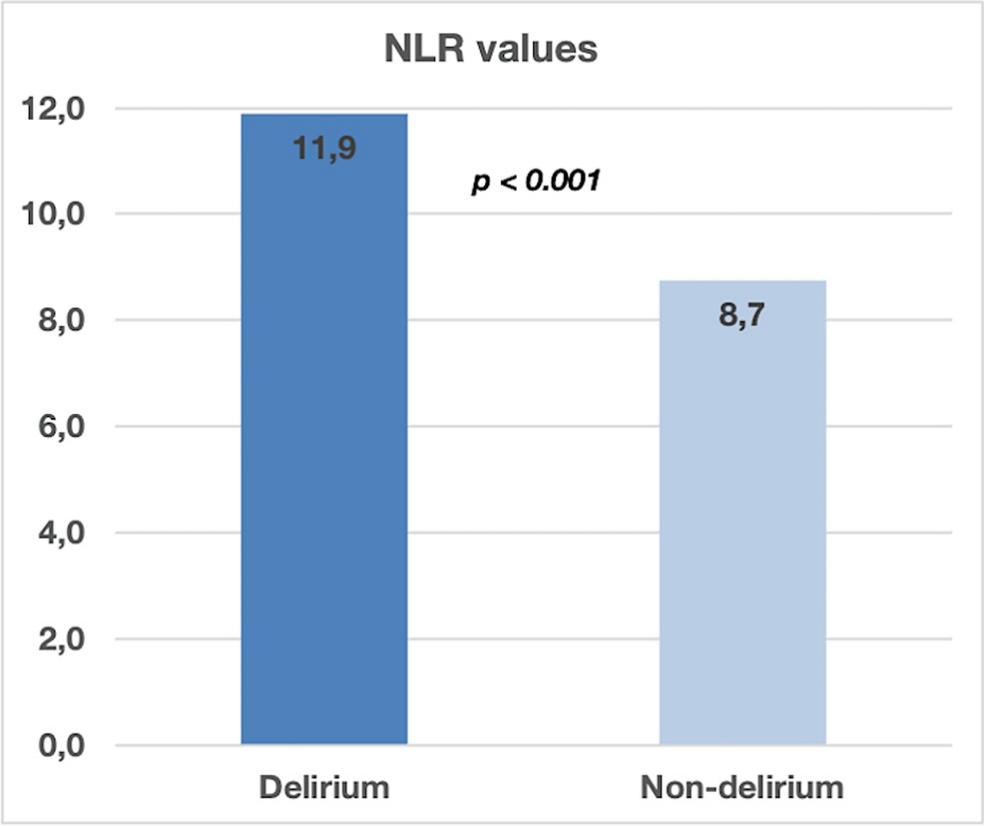
Comparison between septic patients diagnosed with delirium vs non-delirium (p < 0.001) NLR - Neutrophil-to-Lymphocyte Ratio

The only statistically significant relation with the presence of delirium was identified for NLR (Neutrophil-to-Lymphocyte Ratio). The overall value of NLR was 10.3 with significantly higher values for patients with delirium (11.9 vs 8.7, p < 0.001) (Figure 1).

**Figure 2:**
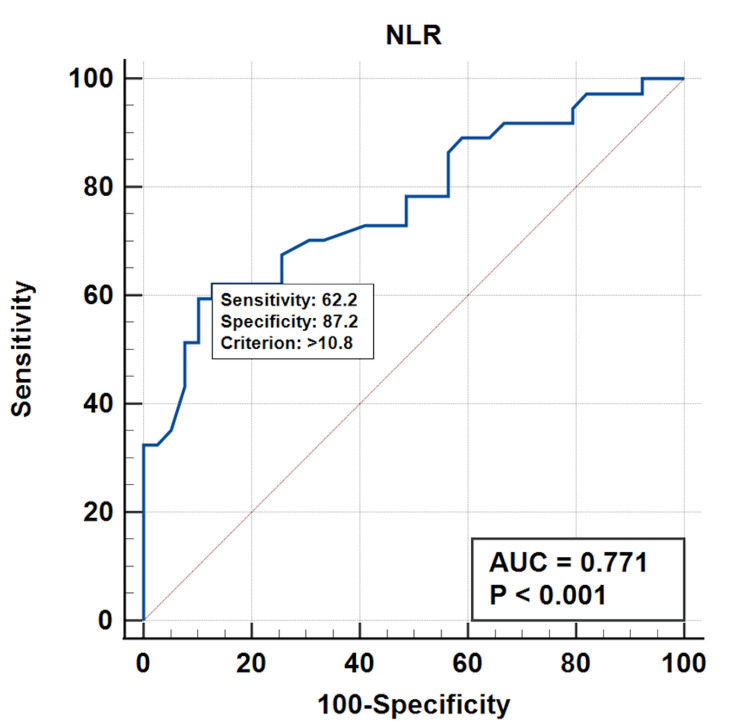
ROC curve for NLR ROC - receiver operating characteristic, NLR - Neutrophil-to-lymphocyte ratio

In order to evaluate the delirium diagnostic performance for NLR, we performed the ROC (receiver operating characteristic) curve analysis (Figure 2). The results showed an area under the curve (AUC) of 0.771 with a rather modest 62.2% sensitivity but a very good specificity of 87.2% for an NLR cut-off value of 10.8 (p<0.001). These results seem to indicate that NLR may serve as a valuable biomarker for early detection of urosepsis-associated delirium. It is therefore possible that delirium occurrence in septic patients may be related to the neurologic inflammatory status from sepsis.

## Discussion

Sepsis, a dysregulated host response to infection, continues to pose a considerable burden on healthcare systems globally. Its prevalence and associated complications are particularly pronounced in the ICU, where critically ill patients are susceptible to severe infections and subsequent organ dysfunction. Among the myriad of complications, delirium emerges as a common neurological manifestation in septic patients, further complicating their clinical course [8]. Sepsis-associated delirium is an issue often seen in patients experiencing organ dysfunctions related to infections. It is believed to result from a mix of neuroinflammation and disruptions in the blood-brain barrier. The exact mechanisms behind sepsis associated delirium are not fully understood yet. It is thought to involve a combination of neuroinflammation, cerebral perfusion issues, the blood-brain barrier, and neurotransmitter activity [1].

Delirium is highly prevalent in septic patients admitted to the ICU, with reported incidence rates ranging from 50% to 80%. Its occurrence is associated with prolonged ICU and hospital stays, increased mortality, and long-term cognitive impairment. Various risk factors contribute to delirium development in sepsis, including advanced age, pre-existing cognitive impairment, severity of illness, use of sedatives and opioid analgesics, and presence of comorbidities [2,4].

In 2022, Dutta et al. in a systematic review emphasises the commonest presentation of urinary tract infection (UTI) in older adults and showed a valid relationship between delirium and UTI. Delirium is a common atypical clinical presentation of UTI in the older population. Delirium can be precipitated by UTI, or UTI can occur due to impaired maintenance of personal hygiene as a result of delirium, leading to unfavourable outcomes [21].

In 2022, Carter and Underwood in an extensive review showed that sepsis-associated delirium (SAD) is a heterogenous condition due to inconsistency in culprit pathogens, pathophysiology and treatment. It remains a common, acute complication of sepsis with chronic sequelae, underlining that the brain may be severely affected during infection [22].

In our study, the incidence of sepsis-associated delirium was 48.7% (37/76 patients). Most of the patients were males (68.4%, 52/76 subjects), but no significant difference was found between the two sexes regarding the presence of delirium. Patient age was significantly higher for those with delirium (72.7 vs 54.5 years, p < 0.001). Also, we found that there were significantly more patients with delirium among the patients with septic shock than those without (29.7% vs 10.2%, p <0.05). Delirium is a common diagnosis in hospitalized elderly patients [12] because of the immune imbalance in elderly patients with sepsis-associated delirium (SAD). Increased serum inflammatory levels of cytokines in SAD suggest this can be the underlying mechanism causing delirium in elderly patients [23].

Various recent studies, including large RCTs, have analysed the impact of the different types of anaesthesia on postoperative delirium incidence after major surgery without finding a consensus. However, all of them evaluated major surgery with prolonged duration of surgery and anaesthesia [24-26].

In our study, endoscopic urological procedures were minimally invasive (ureteral JJ stent placement or replacement, suprapubic or urethro-vesical catheterisation, ultrasound-guided percutaneous nephrostomy or perirenal drainage) and short-timed, with an average duration below minutes. Therefore, the influence of these minimally invasive procedures on cognitive status is insignificant. This is confirmed by several studies showing that prolonged and complex surgical procedures are considered a significant risk factor for postoperative delirium occurrence [27,28].

Recent research has brought attention to the significance of an increased neutrophil, to lymphocyte ratio (NLR) as a marker for delirium linked to urosepsis. NLR is a straightforward and accessible assessment that reflects systemic inflammation and immune response. Zhao et al. in 2021, found that elevated NLR was significantly associated with increased probabilities of delirium in older critical patients [29]. The results suggest that NLR can help as a suitable, low-cost, and fast-accessible marker to predict delirium. The study emphasises that systemic inflammation and oxidative stress play an important role in the pathophysiology of delirium [29]. Higher NLR levels might indicate inflammation and neuro-inflammation making patients more susceptible to delirium in the care unit (ICU) environment [30]. Our results showed that the value of NLR was significantly higher for patients with delirium (11.9 vs 8.7, p < 0.001) so we believe NLR may be a useful tool in early diagnosis of delirium in critically ill patients with urosepsis. Identifying elevated NLR values in critically ill patients dealing with urosepsis and severe infection may lead to targeted actions aimed at preventing confusion from occurring. These strategies could involve treating infections, managing fluids effectively, and reducing risks such as excessive sedation or immobilisation [31].

The practical implications of identifying NLR levels in patients, with sepsis and septic shock are significant. By monitoring NLR levels in urosepsis patients it becomes possible to pinpoint those at a risk of experiencing delirium. Elevated NLR acts as an indicator for results in urosepsis patients, such, as delirium, increased length hospital stays and mortality.

Study limitations and future directions

Limitations of the study include its single-centre setting, lack of dynamic monitoring of NLR values, and relatively small patient sample. Owing to time and cost restraints, we could only enroll a limited number of patients and therefore, we were not able to accurately estimate an adequate patient sample size. Also, due to the scarcity of similar studies, we were unable to find sufficient data to allow us to calculate the sample size needed for the study. Future research should include a more thorough sample size estimation to enhance the accuracy of the findings. Prospective, multi-centre studies with larger cohorts are warranted to further validate the findings and elucidate the mechanisms underlying the association between NLR and urosepsis-associated delirium in the ICU.

## Conclusions

The neutrophil-to-lymphocyte ratio (NLR) is a promising biomarker for the rapid diagnosis and guiding of therapeutic interventions in urosepsis-associated delirium in critically ill patients. Elevated NLR levels seem to be significantly associated with the risk of delirium development and adverse clinical outcomes in the ICU setting.
